# Apoptosis and Cell Cycle Dysregulation in Ampligo^®^ 150 ZC-Induced Nephrotoxicity in Female Rabbits: Protective Effects of *Thymus vulgaris* Essential Oil and Vitamin C

**DOI:** 10.3390/jox16030074

**Published:** 2026-04-27

**Authors:** Louisa Bechohra, Chahrazed Makhlouf, Hassina Khaldoun, Samira Aouichat, Amina Settar, Dalila Tarzaali, Nacera Lemlikchi, Amina Bouhallel, Yasmine Oularbi, Schahinez Terkmane, Nacima Djennane

**Affiliations:** 1 Laboratory of Cellular and Molecular Biology-Tamayouz (Biochemistry Team), Faculty of Biological Sciences, University of Sciences and Technology Houari Boumediene, P.O. Box 32 El Alia, Bab Ezzouar, Algiers 16111, Algeria; 2 Department of Biology, Faculty of Nature and Life Sciences, University of Blida 1, Route de Soumaa, P.O. Box 270, Blida 09000, Algeria; makhlouf_chahrazed@univ-blida.dz (C.M.); khaldoun_hassina@univ-blida.dz (H.K.); 3 Team of Cellular and Molecular Physiopathology, Laboratory of Biology and Physiology of Organisms-Tamayouz, Faculty of Biological Sciences, University of Sciences and Technology Houari Boumediene, P.O. Box 32 El Alia, Bab Ezzouar, Algiers 16111, Algeria; samira_aouichat@outlook.fr; 4 Department of Agri-Food, Faculty of Nature and Life Sciences, University of Blida 1, Route de Soumaa, P.O. Box 270, Blida 09000, Algeria; 5 Institute of Veterinary Sciences, Faculty of Nature and Life Sciences, University of Blida 1, Route de Soumaa, P.O. Box 270, Blida 09000, Algeria; 6 Department of Anatomy, Cytology and Molecular Pathology, Mohamed-Lamine Debaghine University Hospital Center, Bab El Oued, Algiers 16008, Algerianacimadjennane@yahoo.fr (N.D.); 7 Department of Agricultural and Forestry Zoology, Higher National School of Agronomy (ENSA), Algiers 16200, Algeria; 8 Laboratory of Plant Biotechnologies and Ethnobotany, Faculty of Nature and Life Sciences, University of Bejaia, Bejaia 06000, Algeria; terkmane.schahinez@gmail.com

**Keywords:** lambda-cyhalothrin, chlorantraniliprole, nephrotoxicity, p53, apoptosis, E-cadherin, β-catenin, *Thymus vulgaris* essential oil, vitamin C

## Abstract

The widespread use of modern insecticide formulations underscores the need for mechanistic evaluation of their potential renal toxicity. This study investigated the nephrotoxic effects of Ampligo^®^ 150 ZC, a binary formulation of lambda-cyhalothrin and chlorantraniliprole, in female rabbits under subacute exposure conditions, with particular emphasis on apoptosis-related and epithelial integrity biomarkers, and evaluated the protective effects of thyme essential oil (TEO) and vitamin C. Rabbits were allocated into four groups: control, AP, AP + TEO, and AP + TEO + vitamin C. Ampligo (AP) exposure resulted in significant renal dysfunction, as evidenced by elevated biochemical biomarkers and marked histopathological lesions. At the molecular level, AP induced p53 upregulation alongside Bcl-2 and Cyclin D1 downregulation, suggesting apoptosis induction and cell cycle dysregulation. Moreover, reduced E-cadherin and β-catenin expressions indicated disruption of epithelial junction integrity and impaired renal structural homeostasis. Notably, co-administration of TEO and vitamin C markedly attenuated these alterations, improving biochemical, histopathological, and immunohistochemical parameters. Overall, these findings suggest that AP-driven nephrotoxicity may involve apoptotic and epithelial pathways under subacute exposure conditions, whereas antioxidant co-treatment may mitigate kidney injury, supporting the potential of natural antioxidants as adjuncts against pesticide-induced renal injury.

## 1. Introduction

Pesticides are widely used to boost agricultural productivity but pose substantial risks to human health. Exposure occurs primarily through ingestion of contaminated food, inhalation, and dermal contact, with dietary intake representing the main source of systemic exposure [1,2]. This issue is particularly critical for agricultural workers and residents living near farming areas, who are at greater risk of developing adverse health effects, including neurotoxicity, cancer, reproductive disorders, and genotoxic damage [2,3,4,5,6].

One of the critical health concerns associated with pesticide exposure is nephrotoxicity, or kidney damage. Certain insecticides, such as organophosphates and herbicides like atrazine and pendimethalin, have been linked to impaired kidney function in various studies [7,8]. Pesticides can differentially affect both the renal cortex and medulla, although the medulla may exhibit heightened sensitivity to certain toxicants due to its unique metabolic and osmotic environment [9,10,11].

The renal cortex is primarily responsible for glomerular filtration and the initial stage of tubular reabsorption and secretion. It is also involved in metabolizing xenobiotics, particularly in proximal tubular cells where cytochrome P450 enzymes are abundantly expressed [11,12,13]. In contrast, the renal medulla plays a crucial role in urine concentration and fluid electrolyte balance through the loops of Henle and collecting ducts, which is vital for blood pressure regulation [11,14].

Ampligo^®^ 150 ZC (AP) is a novel insecticidal formulation that combines two active ingredients from distinct chemical classes: chlorantraniliprole (9.3%), an anthranilic diamide, and lambda-cyhalothrin (4.6%), a type II synthetic pyrethroid. It has demonstrated broad-spectrum effectiveness against various agricultural pests [15,16,17]. However, AP has also been reported to exhibit significant toxicity to non-target organisms, including annelids, and has been associated with reproductive impairments in both male and female rabbits [18,19].

Chlorantraniliprole (CHL) exerts its insecticidal action by selectively activating insect ryanodine receptors (RyRs) with high affinity, disrupting calcium homeostasis in muscle tissues and causing lethal paralysis in lepidopteran species, while exhibiting approximately 100-fold lower affinity for mammalian RyR1, rendering it purportedly inactive toward mammalian systems [20,21]. Nevertheless, accumulating evidence indicates that CHL can induce adverse effects in mammalian models, including anxiety-like behaviors, neuronal activation in the hippocampus, developmental and genetic toxicity, reproductive impairment, and renal dysfunction accompanied by anemia [22,23].

Lambda-cyhalothrin (LCT), a widely used type II pyrethroid, targets voltage-gated sodium channels in insects, disrupting neuronal excitability and causing paralysis [24,25]. Although effective against a broad range of pests, LCT has been shown to elicit toxic effects in non-target organisms [26,27]. In mammalian models, LCT exposure has been associated with genotoxicity, oxidative stress, and histopathological damage in multiple organs, including the brain, liver, thyroid, testes, pancreas, and kidney [28,29,30,31,32,33,34].

The combination of lambda-cyhalothrin and chlorantraniliprole may result in enhanced biological effects due to complementary mechanisms. Lambda-cyhalothrin is known to induce oxidative stress and membrane destabilization, whereas chlorantraniliprole disturbs intracellular calcium homeostasis through ryanodine receptor modulation [19,20,22]. The convergence of these mechanisms may amplify cellular stress responses, particularly in metabolically active organs such as the kidney, potentially increasing susceptibility to tissue injury.

Nutraceuticals and plant-derived bioactive compounds, rich in antioxidants, vitamins, and polyphenols, have shown potential to mitigate pesticide-induced toxicity by supporting detoxification and cellular repair, offering a promising strategy to protect renal health in exposed populations [35,36]. Essential oils obtained from *Thymus vulgaris* L. (TEO), particularly those rich in phenolic compounds such as carvacrol, are widely recognized for their potent antioxidant activity [37,38].

Notably, TEO has demonstrated renoprotective effects in insecticide-exposed models [39]. Similarly, supplementation with vitamins A, C, D, and E, or vitamin C alone, has been shown to ameliorate hepato-renal toxicity in rabbits and rats by preventing oxidative damage to biological molecules [29,40,41].

To the best of our knowledge, studies investigating the nephrotoxic effects of the combined active compounds chlorantraniliprole and lambda-cyhalothrin in the newly formulated insecticide Ampligo^®^ 150 ZC are lacking. In addition, the potential differences between the renal cortex and medulla following pesticide exposure remain poorly characterized. Furthermore, the co-treatment of plant-derived polyphenols and vitamins as a protective strategy against pesticide-induced nephrotoxicity has not been sufficiently explored.

Therefore, the present study investigated the subacute nephrotoxicity of Ampligo^®^ 150 ZC in female rabbits using a multi-level approach integrating histopathological, biochemical, and immunohistochemical analyses. Particular emphasis was placed on markers related to apoptosis, cell cycle regulation, and epithelial integrity (p53, Bcl-2, Cyclin D1, E-cadherin, and β-catenin). In addition, this study aimed to evaluate the potential protective effects of *Thymus vulgaris* essential oil (TEO), characterized by its high carvacrol content [39], when co-administered with vitamin C, on cortico-medullary renal alterations induced by pesticide exposure.

## 2. Materials and Methods

### 2.1. Insecticide and Chemicals

The commercial insecticide Ampligo^®^ 150 ZC (AP) is a formulated mixture containing chlorantraniliprole (C_18_H_14_BrCl_2_N_5_O_2_; 100 g/L; CAS No. 500008-45-7) and lambda-cyhalothrin (C_23_H_19_ClF_3_NO_3_; 50 g/L; CAS No. 91465-08-6). The product was obtained from Syngenta Crop Protection AG (Basel, Switzerland). Vitamin C (ascorbic acid; catalog No. 1004681000) was purchased from Sigma Chemical Co (Saint-Quentin-Fallavier, France).

### 2.2. Thyme Essential Oil

Thyme essential oil (TEO) was extracted from the aerial parts of Algerian thyme (*Thymus vulgaris* L.; Lamiaceae), collected during the flowering season in June 2020 in Blida Province (Hammam Melouane, northern Algeria; 36°29′ N, 2°50′ E; altitude: 200 m). The plant material was identified by botanists at the National Higher School of Agronomy, Algiers, Algeria. TEO was obtained by hydrodistillation using a Clevenger-type apparatus and dried over anhydrous sodium sulfate (Na_2_SO_4_). Before administration, the essential oil was dissolved in olive oil. The chemical composition of the TEO used in the present study was previously characterized by our research group using GC–MS analysis, revealing carvacrol as the major constituent (86.25% of total volatile compounds) [39].

### 2.3. Animals

A total of 20 female rabbits (*Oryctolagus cuniculus*), aged 5 months and weighing 2–3 kg, were obtained from the Technical Breeding Institute (ITLEV Baba Ali, Algeria) and housed at Saad Dahlab Blida 1 University for the experiments. The rabbits were acclimatized for two weeks in individual metal cages under controlled conditions (22 ± 2 °C, 45–65% humidity, and a 12 h light/dark cycle). They were provided with a standard commercial pellet diet and water ad libitum.

### 2.4. Experimental Study Design

The rabbits were randomly assigned into four groups (*n* = 5 per group). The control group received 1 mL of distilled water by oral gavage. The second group (AP) received Ampligo^®^ alone at a dose of 20 mg/kg body weight, administered orally every other day for 28 days. The selected dose was based on previous experimental studies and was intended to induce measurable toxicological effects under subacute exposure conditions. The third group (AP + TEO) received thyme essential oil (0.5 mg/kg body weight) by oral gavage in combination with the same dose of Ampligo^®^. The fourth group (AP + TEO + Vit C) received vitamin C (200 mg/kg body weight) by oral gavage, followed two hours later by thyme essential oil and Ampligo^®^ at the same doses [19,39].

Throughout the acclimatization and experimental periods, body weight, food intake, and water consumption were monitored daily. The experimental design was focused on evaluating the protective effect of TEO alone and in combination with vitamin C against AP-induced nephrotoxicity; therefore, a vitamin C-only group was not included. The experimental design was conducted in accordance with OECD guidelines for repeated-dose toxicity studies (28-day exposure) and in compliance with ARRIVE guidelines. The study was approved by the Institutional Animal Care Committee under the National Administration of Algerian Higher Education and Scientific Research (DE n°10–90).

### 2.5. Evaluation of Renal Function

At the conclusion of the 28-day experiment, blood samples were collected to measure serum levels of renal parameters: creatinine, blood urea and uric acid using the Hitachi 912 Clinical Chemistry Analyzer (Hitachi, Ltd., Tokyo, Japan).

### 2.6. Histomorphometrtical Analysis

At the end of the experiment, rabbits were euthanized in accordance with ethical guidelines, and kidneys were excised, weighed, and fixed in 10% neutral-buffered formalin for 48 h. Fixed tissues were dehydrated through a graded ethanol series, cleared in xylene, and embedded in paraffin. Serial sections (2–3 μm thick) were cut using a Leica RM2125 RTS microtome (Leica Biosystems Nussloch GmbH, Nussloch, Germany) and stained with hematoxylin and eosin (H&E) for histopathological examination.

To complement the qualitative histological observations, renal lesions were also assessed using a semi-quantitative histomorphometric scoring system. Six random fields per kidney were analyzed at 400× magnification using light microscopy (Optika B-183, Ponteranica, Bergamo, Italy). Corticomedullary injury was scored on a scale from 0 to 4 according to the percentage of damaged renal tubules: 0 (no damage), 1 (<25%), 2 (26–50%), 3 (51–75%), and 4 (≥76%), as previously described [42]. In addition, Masson’s trichrome staining was performed to evaluate renal fibrosis, and the collagen-positive area was quantified using ImageJ software [43].

### 2.7. Immunohistochemical Assessment

Immunohistochemistry was performed on formalin-fixed, paraffin-embedded kidney sections using an automated staining system (Benchmark ULTRA; Ventana Medical Systems, Tucson, AZ, USA), according to the manufacturer’s instructions. The primary antibodies used were p53 (clone Bp–53–11, cat# 760–2542), Cyclin D1 (clone SP4–R, cat# 790–4508), Bcl-2 (clone SP66, cat# 790–4604), β-catenin (clone 14, cat# 760–4242), and E-cadherin (clone EP700Y, cat# 760–4440).

Positive immunostaining was quantified using ImageJ software (version 1.49; NIH, Bethesda, MD, USA) with a color deconvolution plugin and a fixed threshold. Results were expressed as the percentage of positive staining area within a standardized measurement frame and analyzed at 400× magnification.

### 2.8. Statistical Analysis

All data are presented as mean ± standard deviation. Normality and homogeneity of variance were assessed using the Shapiro–Wilk test. Statistical analyses were performed using one-way analysis of variance (ANOVA), followed by Tukey’s post hoc test for multiple comparisons between groups. Data analysis was conducted using GraphPad Prism version 8.1 (GraphPad, Inc., CA, USA). A *p*-value < 0.05 was considered statistically significant.

## 3. Results

### 3.1. Effects of TEO and/or Vitamin C on Body Weights and Kidneys Weights in Insecticide-Treated Rabbits

Rabbits exposed to AP and the different treatment regimens were closely monitored throughout the experimental period, and no mortality was recorded. A significant decrease in mean body weight was observed in the AP-treated group from the second week of exposure (*p* < 0.05), and this reduction persisted until the end of the 28-day experimental period (*p* < 0.01) compared with the control group (Table 1). Co-treatment with TEO and/or vitamin C significantly attenuated body weight loss compared with the AP-treated group (*p* < 0.01). Similarly, treatment with TEO and/or vitamin C markedly alleviated the reduction in both absolute and relative kidney weights observed in AP-exposed female rabbits. Among the treatment groups, the co-administration of TEO and vitamin C showed the greatest protective effect on body weight and kidney weight restoration (*p* < 0.01) compared with TEO alone (Table 1).

### 3.2. Effects of TEO and/or Vitamin C on Renal Function in Insecticide-Treated Rabbits

Renal function was evaluated by measuring serum levels of the principal renal biomarkers, namely blood urea nitrogen (BUN), creatinine, and uric acid, as shown in Table 2. After 28 days of exposure, AP-treated rabbits exhibited significant increases in all three biomarkers compared with the control group (*p* < 0.05; *p* < 0.01), indicating impaired renal function. Co-treatment with TEO alone and, more prominently, in combination with vitamin C significantly attenuated these alterations. More specifically, TEO treatment reduced BUN, creatinine, and uric acid levels by 31.82%, 12.76%, and 55.34%, respectively, whereas the co-treatment reduced them by 34.1%, 15.1%, and 65.91%, respectively. These findings indicate an improvement in renal function in treated rabbits (*p* < 0.01).

### 3.3. Protective Effects of TEO and/or Vitamin C on Insecticide-Induced Renal Histopathological Changes

**Figure 1:**
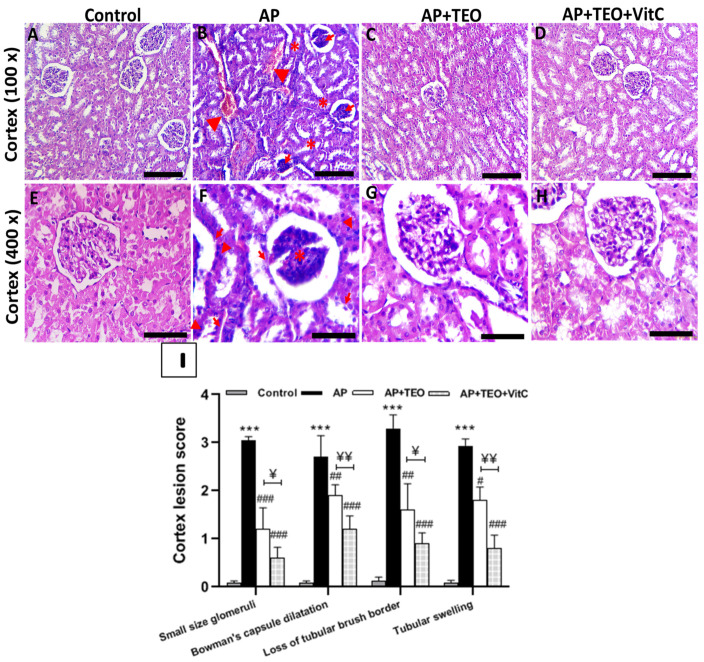
Protective effects of Thyme Essential Oil (TEO) and/or Vitamin C on histopathological alterations induced by Ampligo exposure in renal cortex rabbit tissue sections. (**A**) The control group shows normal glomerular structure and well-organized renal tubules. (**B**) Glomerular shrinkage and early signs of sclerosis (→), associated with vascular congestion (▲) and tubular disorganization (*) were observed in the AP group. (**C**,**D**) The TEO- and AP + TEO + VitC-treated groups exhibit preserved glomerular morphology and tubular integrity, with no visible signs of congestion or major structural disruption. (**E**) At higher magnification, the control group reveals normal glomeruli structure and intact tubular structures with preserved epithelial morphology. (**F**) In the AP group, increased glomerular matrix deposition (*), tubular epithelial desquamation and loss of proximal tubular brush border (→), as well as increased apoptotic nuclei (▲) are evident. (**G**,**H**) These lesions are markedly attenuated in the TEO- and TEO + VitC-treated groups, which display improved epithelial morphology and reduced glomerular structural damage. (**I**) Semi-quantitative evaluation of the histo-pathological lesions. *** *p* < 0.001 compared with the control group; ## *p* < 0.01; ### *p* < 0.001 compared with the insecticide-treated group. ^¥^ *p* < 0.05; ^¥¥^ *p* < 0.01 indicates statistical significance between AP + TEO and AP + TEO + VitC groups. Top panels (**A**–**D**): original magnification at 100×; bottom panels (**E**–**H**): original magnification at 400×. Sections stained with hematoxylin and eosin (H&E). Scale bars: 400 µm (100×); 150 µm (400×).

In the present study, histopathological evaluation of renal tissue was performed in insecticide-exposed rabbits, with and without treatment with TEO and/or vitamin C. Hematoxylin and eosin staining was used to assess morphological alterations (Figure 1). Renal cortex sections from the control group exhibited preserved histological architecture (Figure 1A,E). In contrast, tissue sections from AP-treated rabbits showed marked pathological alterations, including glomerular atrophy with early sclerotic changes, vascular congestion, tubular disorganization, and an increased number of pyknotic nuclei (Figure 1B,F). These histopathological lesions were markedly attenuated in the groups treated with TEO (Figure 1C,G) and with the co-treatment TEO + vitamin C treatment (Figure 1D,H). In both groups, epithelial integrity was largely preserved, and glomerular structural damage was clearly reduced.

To complement the representative histological observations, renal lesions were also evaluated using a semi-quantitative histomorphometric scoring system. Histomorphometric analysis of cortical lesions in rabbit kidneys revealed a significant increase in overall lesion scores in the AP-treated group compared with the control group (*p* < 0.001). Higher scores reflected reductions in glomerular size, dilation of Bowman’s capsule, tubular swelling, and loss of brush border integrity. Co-treatment with thyme essential oil (TEO) and/or vitamin C significantly reduced lesion scores across all evaluated parameters (*p* < 0.01). Notably, the co-treatment with TEO and vitamin C produced a significantly greater protective effect than TEO alone, as reflected by lower lesion scores (*p* < 0.01) (Figure 1I).

**Figure 2:**
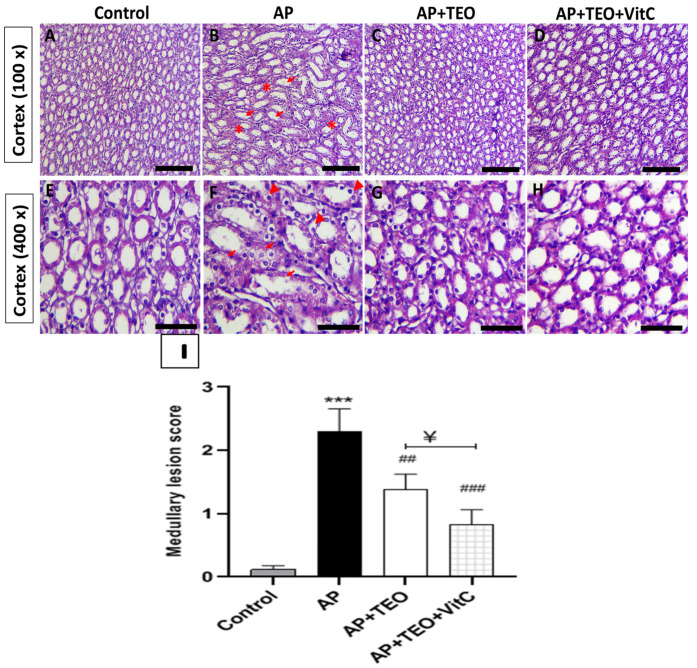
Protective effects of Thyme Essential Oil (TEO) and/or Vitamin C on histopathological alterations induced by Ampligo exposure in renal medulla rabbit tissue sections. (**A**) The control group displays normal organization of medullary tubules and collecting ducts with uniform structure. (**B**) In the AP group, altered tissue architecture is visible, with tubular disorganization (→) and luminal dilatation (*). (**C**,**D**) The TEO- and TEO +VitC-treated groups display improved tubular arrangement. (**E**) At higher magnification, control group exhibits intact epithelial lining and regular luminal profiles. (**F**) The AP group shows tubular epithelial degeneration, with loss of epithelial cells (▲), and flattened or detached epithelium (→). (**G**,**H**) These lesions are attenuated in the TEO- and TEO + VitC-treated groups, which exhibit partial recovery of epithelial integrity and more preserved tubular morphology. (**I**) Semi-quantitative evaluation of the histo-pathological lesions. *** *p* < 0.001 compared with the control group; ## *p* < 0.01; ### *p* < 0.001 compared with the insecticide-treated rabbits. ^¥^ *p* < 0.05 indicates statistical significance between AP + TEO and AP + TEO + VitC groups. Top panels (**A**–**D**): original magnification at 100×; bottom panels (**E**–**H**): original magnification at 400×. Sections stained with hematoxylin and eosin (H&E). Scale bars: 400 µm (100×); 150 µm (400×).

As shown in Figure 2, H&E staining of medullary tissue revealed that the medullary tubules and collecting ducts in the control group displayed normal histological architecture with a uniform structural appearance (Figure 2A,E). In contrast, the AP-treated group exhibited pronounced pathological alterations, particularly in the collecting ducts and loops of Henle within the outer medulla (Figure 2B,F). These changes were characterized by epithelial cell loss, luminal dilatation, and a flattened or detached epithelial lining. Most of these medullary alterations were markedly attenuated following co-treatment with TEO and/or vitamin C (Figure 2C,D,G,H).

Histomorphometric analysis of the medullary region showed a significant increase in outer medullary lesion scores in the AP-treated group compared with the control group (2.30 ± 0.35 vs. 0.11 ± 0.06, *p* < 0.001) (Figure 2I). In addition, the co-treatment exerted a greater protective effect than TEO alone, as reflected by lower lesion scores (0.82 ± 0.23 vs. 1.37 ± 0.25, *p* < 0.05) (Figure 2I).

### 3.4. Effects of TEO and/or Vitamin C on Fibrosis in the Renal Cortico-Medullary Tissue of Ampligo -Treated Rabbits

**Figure 3:**
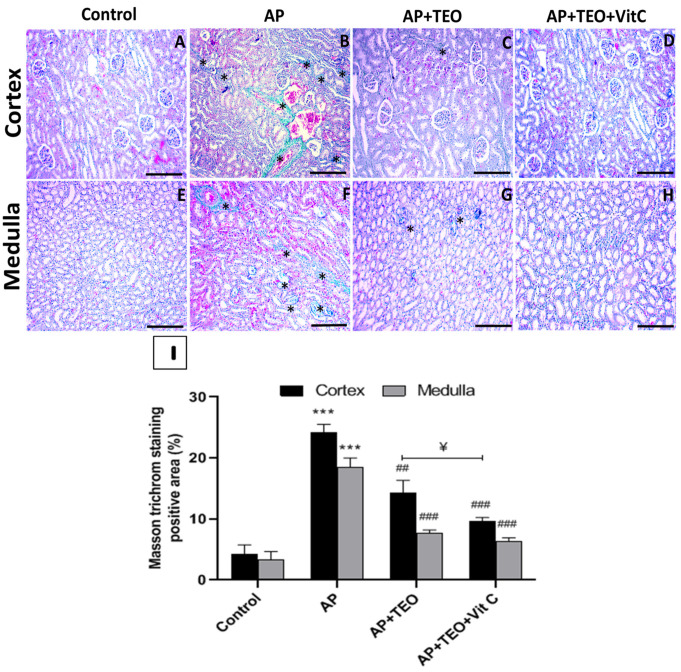
Masson’s trichrome staining showing collagen deposition in the cortical and medullary regions of rabbit kidneys from the different experimental groups, observed under light microscopy at 100× magnification. Control group (**A**,**E**), AP-treated group (**B**,**F**), AP + TEO group (**C**,**G**), and AP + TEO + vitamin C group (**D**,**H**). (*) indicate the areas used for collagen quantification in panels (**B**,**C**,**F**,**G**). (**I**) Collagen deposition (*) was quantified using ImageJ software (NIH, Bethesda, MD, USA) with color deconvolution to isolate collagen-specific staining in 10 randomly selected fields from cortical and medullary sections. Scale bars: 400 µm. Data are expressed as mean ± SD (*n* = 5). *** *p* < 0.001 versus the control group; ## *p* < 0.01 and ### *p* < 0.001 versus the AP-treated group. ^¥^ *p* < 0.05 indicates statistical significance between the AP + TEO and AP + TEO + vitamin C groups.

As illustrated in Figure 3B,F, AP-treated rabbits exhibited a significant increase in Masson’s trichrome-positive areas within the tubulointerstitial compartments and peritubular capillaries of both the cortex and medulla, suggesting increased collagen deposition and fibrosis. Treatment with TEO (0.5 mg/kg) markedly attenuated these alterations, as reflected by significantly lower fibrotic scores in the cortex (14.3 ± 1.66% vs. 24.19 ± 1.06%, *p* < 0.01) and medulla (7.73 ± 0.37% vs. 18.5 ± 1.22%, *p* < 0.001) (Figure 3C,G,I). Moreover, the co-treatment was associated with a greater reduction in cortical fibrosis than TEO alone (9.66 ± 0.47% vs. 14.3 ± 1.66%, *p* < 0.05), whereas the difference in the medulla was not statistically significant (6.33 ± 0.47% vs. 7.73 ± 0.35%) (Figure 3D,H,I). These findings indicate that co-treatment with TEO and vitamin C attenuated renal tubulointerstitial fibrosis in AP-exposed rabbits.

### 3.5. Effects of TEO and Vitamin C on p53 and Bcl-2 Immunoreactivity in the Kidneys of Ampligo-Exposed Rabbits

**Figure 4:**
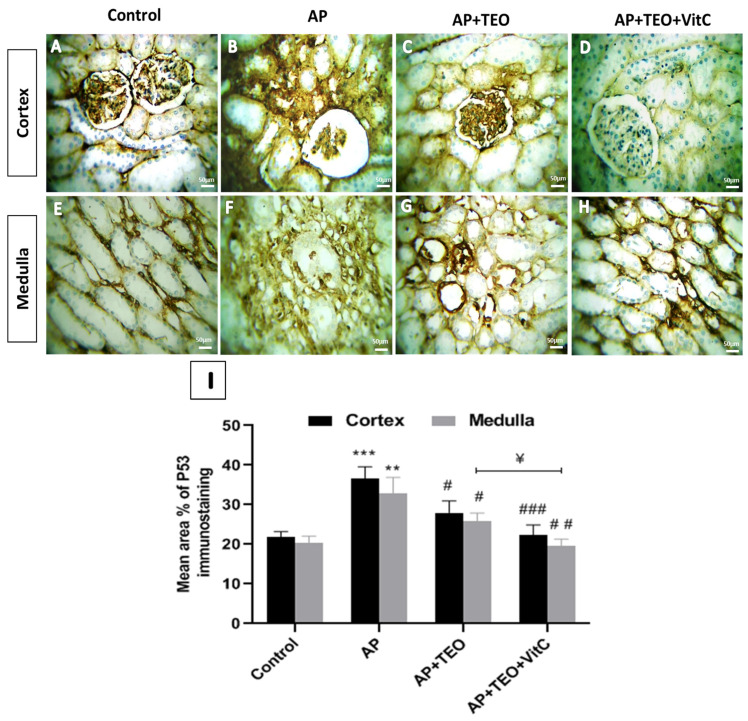
Immunohistochemical staining of p53 in the cortical and medullary regions of female rabbit kidneys. Panels (**A**,**E**) show weak p53 immunostaining in control kidneys. Panels (**B**,**F**) show intense p53 immunoreactivity in the cortical and medullary regions of AP-treated rabbits. Panels (**C**,**G**,**D**,**H**) show reduced p53 staining in the AP + TEO and AP + TEO + vitamin C groups, respectively. Images were obtained at 400× magnification; scale bar = 150 μm. Panel (**I**) shows the morphometric analysis of the percentage area of p53 immunoreactivity in the kidney cortex and medulla. Data are presented as mean ± SD (*n* = 5). ** *p* < 0.01 and *** *p* < 0.001 versus the control group; # *p* < 0.05, ## *p* < 0.01, and ### *p* < 0.001 versus the AP-treated group. ^¥^ *p* < 0.05 indicates statistical significance between the AP + TEO and AP + TEO + vitamin C groups.

Immunohistochemical analysis showed low basal p53 expression in tubular cells of both the cortex and medulla in control kidneys (Figure 4A,E). In contrast, AP-treated rabbits exhibited a significant increase in p53 immunostaining in both cortical (36.70 ± 2.34%) and medullary (32.83 ± 3.32%) tubular cells compared with controls (cortex: 21.93 ± 1.01%; medulla: 20.29 ± 1.47%; *p* < 0.001 for cortex and *p* < 0.01 for medulla) (Figure 4B,F,I). This increased expression was localized in both the nuclei and cytoplasm of proximal tubular cells in the cortex, as well as in cells of the thick ascending limb and collecting ducts in the medulla. Co-treatment with TEO (Figure 4C,G) and with TEO + vitamin C (Figure 4D,H) significantly reduced p53 immunoreactivity (*p* < 0.001), restoring its expression in both regions toward control levels. Notably, in the medulla, the co-treatment with TEO and vitamin C produced a significantly greater reduction in p53 expression than TEO alone (*p* < 0.05).

**Figure 5:**
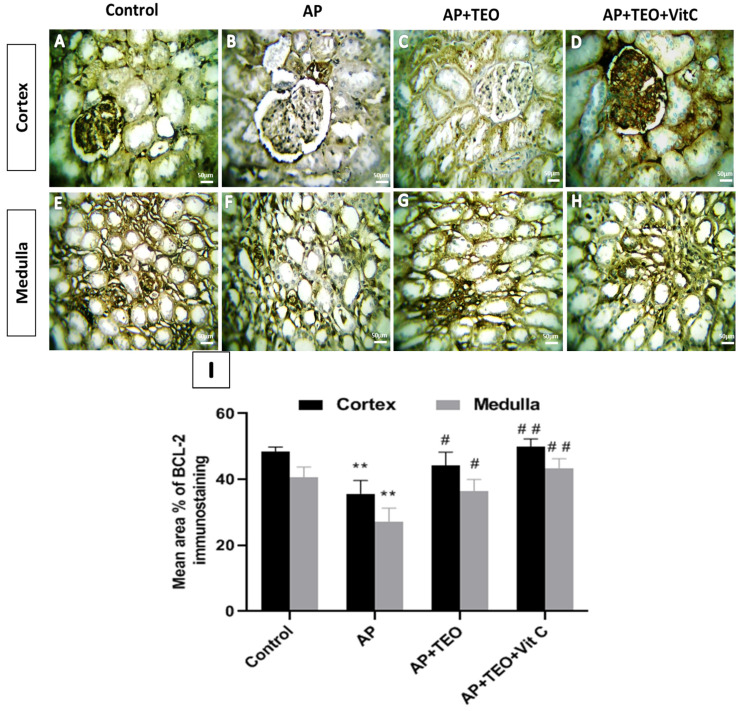
Immunohistochemical staining of Bcl-2 in the cortical and medullary regions of female rabbit kidneys. Panels (**A**,**E**) show marked Bcl-2 immunostaining in control kidneys. Panels (**B**,**F**) show reduced Bcl-2 immunoreactivity in the AP-treated group, whereas panels (**C**,**G**,**D**,**H**), show preserved Bcl-2 expression in the AP + TEO and AP + TEO + vitamin C groups, respectively. Images were obtained at 400× magnification; scale bar = 150 μm. Panel (**I**) shows the morphometric analysis of the percentage area of Bcl-2 immunoreactivity in the kidney cortex and medulla. Data are presented as mean ± SD (*n* = 5). ** *p* < 0.01 versus the control group; # *p* < 0.05 and ## *p* < 0.01 versus the AP-treated group.

Bcl-2 immunostaining showed diffuse cytoplasmic expression in tubular epithelial cells of both cortical and medullary regions in control kidneys (Figure 5A,E). AP treatment resulted in a significant reduction in Bcl-2 immunoreactivity in both the cortex (35.66 ± 3.25%) and medulla (27.08 ± 3.47%) compared with controls (cortex: 48.48 ± 1.11%; medulla: 40.59 ± 2.53%; *p* < 0.01) (Figure 5B,F,I). In contrast, Bcl-2 expression in the AP + TEO (Figure 5C,G) and AP + TEO + vitamin C (Figure 5D,H) groups remained comparable to control values, with no significant differences observed in either cortical or medullary regions (Figure 5I).

### 3.6. Modulation of Cyclin D1 Expression by TEO and/or Vitamin C During Ampligo-Induced Nephrotoxicity

**Figure 6:**
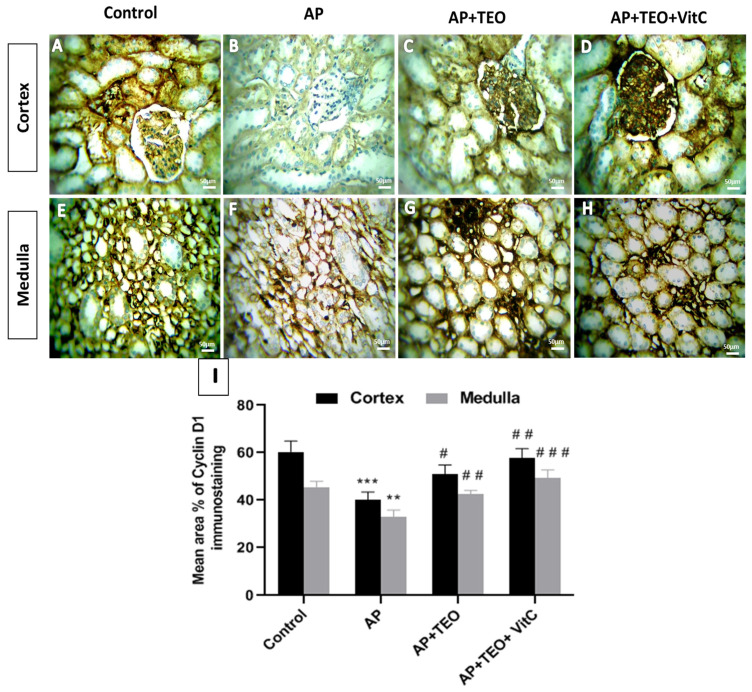
Immunohistochemical staining of Cyclin D1 in the cortical and medullary regions of female rabbit kidneys. Panels (**A**,**E**) show strong Cyclin D1 immunostaining in control kidneys. Panels (**B**,**F**) show reduced Cyclin D1 immunoreactivity in the AP-treated group. Panels (**C**,**G**,**D**,**H**), show preserved Cyclin D1 expression in the AP + TEO and AP + TEO + vitamin C groups, respectively. Images were obtained at 400× magnification; scale bar = 150 μm. Panel (**I**) shows the morphometric analysis of the percentage area of Cyclin D1 immunoreactivity in the kidney cortex and medulla. Data are presented as mean ± SD (*n* = 5). ** *p* < 0.01 and *** *p* < 0.001 versus the control group; # *p* < 0.05, ## *p* < 0.01, and ### *p* < 0.001 versus the AP-treated group.

To further explore the mechanisms underlying the protective effects of TEO and/or vitamin C against Ampligo-induced nephrotoxicity, Cyclin D1 expression was evaluated by immunohistochemistry in rabbit kidney tissue (Figure 6). As shown in Figure 6A,E, Cyclin D1 immunostaining was markedly reduced in the renal cortex and medulla of AP-treated rabbits, with weaker cytoplasmic expression in tubular epithelial cells (Figure 6B,F). Quantitative analysis revealed a significant decrease in the mean area percentage of Cyclin D1 expression in the AP-treated group (cortex: 40.23 ± 3.21%; medulla: 33.06 ± 2.75%) compared with the control group (cortex: 60.35 ± 4.53%; medulla: 45.59 ± 2.34%), with statistical significance at *p* < 0.001 in the cortex and *p* < 0.01 in the medulla (Figure 6I). In contrast, co-treatment with AP + TEO (Figure 6C,G) or AP + TEO + vitamin C (Figure 6D,H) was associated with preservation of Cyclin D1 expression, with immunostaining levels remaining comparable to those observed in the control group (Figure 6I).

### 3.7. Alteration of E-Cadherin/β-Catenin Expression in Ampligo-Induced Renal Injury and Its Modulation by Antioxidant Treatment

**Figure 7:**
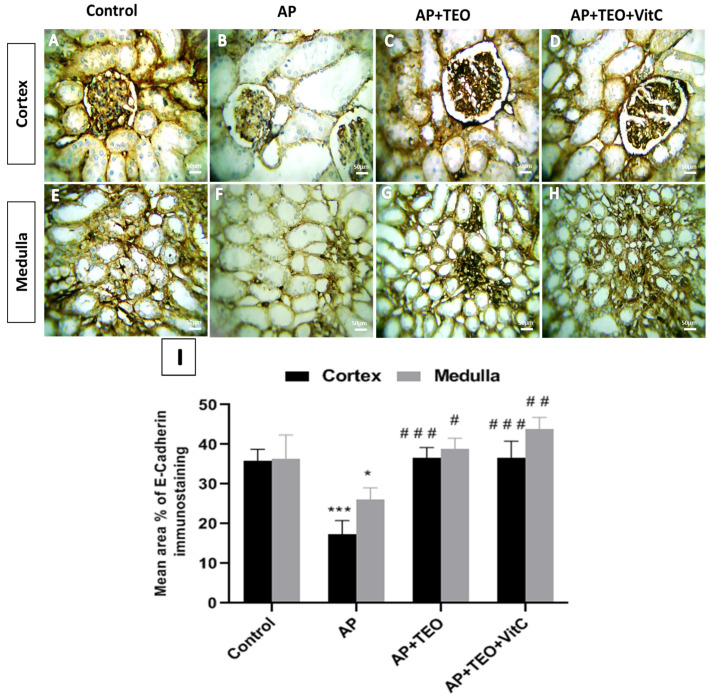
Immunohistochemical staining of E-cadherin in the cortical and medullary regions of female rabbit kidneys. Marked E-cadherin immunostaining was observed in the control group (**A**,**E**), the AP + TEO group (**C**,**G**), and the AP + TEO + vitamin C group (**D**,**H**), whereas reduced immunoreactivity was observed in the AP-treated group (**B**,**F**). All micrographs were captured at 400× magnification; scale bar = 150 μm. Panel (**I**) shows the quantitative morphometric analysis of the percentage area of E-cadherin immunoreactivity in the cortex and medulla. Data are presented as mean ± SD (*n* = 5). * *p* < 0.05 and *** *p* < 0.001 versus the control group; # *p* < 0.05, ## *p* < 0.01, and ### *p* < 0.001 versus the AP-treated group.

E-cadherin membrane expression is shown in Figure 7. In the control group, normal E-cadherin expression was observed in both the cortex and medulla (35.86 ± 2.89% and 36.34 ± 6.03%, respectively) (Figure 7A,E,I). In contrast, AP-treated rabbits exhibited a significant decrease in E-cadherin membrane expression in both the cortex (17.43 ± 3.39%) and medulla (26.03 ± 3.05%) compared with controls (*p* < 0.001) (Figure 7B,F,I). Co-treatment with TEO restored E-cadherin immunoreactivity in both cortical and medullary regions (36.55 ± 2.62% and 38.83 ± 2.72%, respectively). Likewise, the co-treatment with TEO + vitamin C was associated with increased E-cadherin expression in the cortex and medulla (36.68 ± 4.14% and 43.85 ± 3.01%, respectively) compared with the AP-treated group.

**Figure 8:**
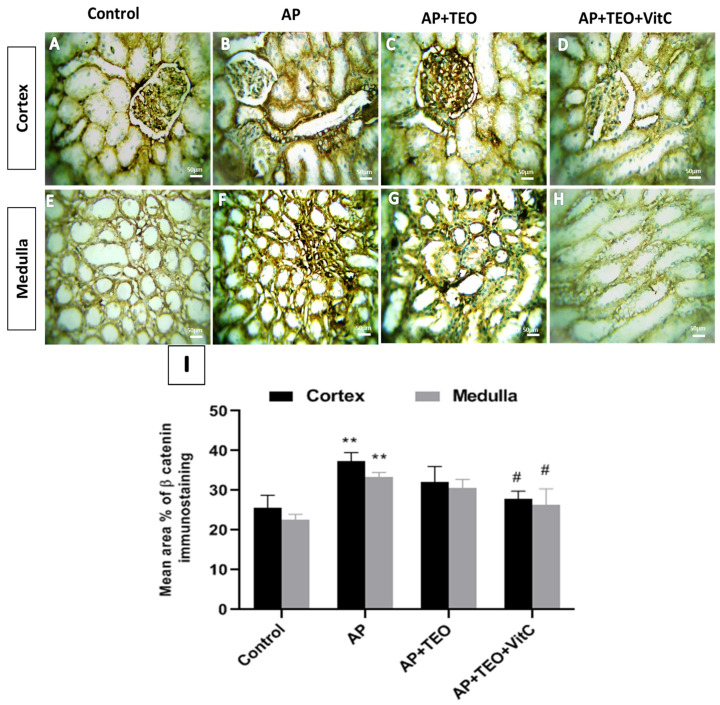
Immunohistochemical staining of β-catenin in the cortical and medullary regions of female rabbit kidneys from the control, AP, AP + TEO, and AP + TEO + vitamin C groups. Panels (**A**,**E**) show moderate membranous β-catenin expression at the cell–cell boundaries of epithelial cells in control kidneys. Panels (**B**,**F**) show intense cytoplasmic and nuclear β-catenin immunoreactivity in the AP-treated group. Panels (**C**,**G**,**D**,**H**), show moderate β-catenin immunoreactivity in both membranous and cytoplasmic compartments of epithelial cells in the cortex and medulla of rabbits treated with AP + TEO and AP + TEO + vitamin C, respectively. All micrographs were captured at 400× magnification; scale bar = 150 μm. Panel (**I**) shows the quantitative morphometric analysis of the percentage area of β-catenin immunoreactivity in the cortex and medulla. Data are presented as mean ± SD (*n* = 5). ** *p* < 0.01 versus the control group; # *p* < 0.05 versus the AP-treated group.

As shown in Figure 8, β-catenin immunostaining in control kidneys was mainly localized at the cell membrane in both cortical and medullary structures (Figure 8A,E). In contrast, AP exposure was associated with a significant increase in β-catenin immunoreactivity in the cytoplasm and nucleus of renal cells in both the cortex (37.29 ± 2.22%) and medulla (33.34 ± 1.16%) compared with controls (Figure 8B,F,I). Interestingly, co-treatment with TEO + vitamin C significantly reduced β-catenin expression in both the cortex (27.91 ± 2.33%) and medulla (26.43 ± 4.06%) compared with the AP-treated group (Figure 8D,H,I).

## 4. Discussion

The kidney is highly susceptible to toxic injury because it receives approximately 20–25% of cardiac output and actively concentrates xenobiotics and their metabolites within tubular epithelial cells [44]. Modern insecticide formulations often combine compounds with distinct molecular targets, raising concerns about additive or possibly combined toxicity, while also increasing environmental persistence and potential human health risks [45]. Agricultural workers represent the most vulnerable population because of repeated and prolonged exposure during pesticide handling and application. Ampligo^®^ 150 ZC (AP) combines lambda-cyhalothrin, a type II pyrethroid that disrupts voltage-gated sodium channels and induces oxidative stress, with chlorantraniliprole, an anthranilic diamide that alters calcium homeostasis through ryanodine receptor activation [46]. Recent studies indicate that such combinations may amplify oxidative stress, mitochondrial dysfunction, inflammatory signaling, and apoptosis in non-target organs, including the kidney [47,48].

Thyme essential oil (TEO) from *Thymus vulgaris* has been reported to exert protective effects against toxic insults involving oxidative stress, inflammation, and cellular injury, which are key mechanisms in pesticide-induced nephrotoxicity [49]. Its phenolic constituents, particularly thymol and carvacrol, are associated with strong antioxidant activity through free-radical scavenging and enhancement of endogenous antioxidant defenses, thereby helping to limit lipid peroxidation and preserve cellular integrity under chemical stress [50]. Vitamin C, a well-established water-soluble antioxidant, may complement these effects by directly neutralizing reactive oxygen species and supporting the re-generation of other antioxidants, which can reduce oxidative damage and apoptosis in renal tissues exposed to toxicants especially pyrethroids [51]. Taken together, these findings support a potential complementary interaction between TEO and vitamin C.

Body weight serves as a global indicator of systemic toxicity in experimental toxicology. In the present study, AP-treated rabbits exhibited a significant body weight reduction compared to controls (*p* < 0.01), whereas co-treatment with TEO and vitamin C partially attenuated this effect. Sustained body weight loss reflects metabolic dysregulation, reduced feed intake, mitochondrial dysfunction or endocrine disruption [52]. These findings suggest AP-induced systemic toxicity likely mediated by oxidative stress impairing metabolic efficiency, consistent with lambda-cyhalothrin studies in rabbits [53]. Antioxidant co-treatment indicates partial restoration of redox balance and metabolic homeostasis [41,54,55], corroborating reports where vitamins and plant phenolics partially or completely recovered body weight in lambda-cyhalothrin-exposed animals [18,19,56].

In our obtained results, AP-exposed rabbits exhibited significant decrease in both absolute and relative kidney weights, consistent with insecticide-induced renal toxicity. Reduced kidney weight typically reflect compromised renal structural integrity and altered energy homeostasis following toxicant exposure [57]. These findings align with Oularbi et al. [30], reporting similar decreases, but contrast with Boumezrag et al. and Bakheet et al. [55,58] who observed kidney weight increases with lambda-cyhalothrin in rabbits/rats. Such discrepancies likely reflect differences in dose, duration, or species-specific responses.

Blood urea nitrogen (BUN), serum creatinine, and uric acid are established indicators of glomerular filtration and tubular function. Elevated BUN and creatinine typically signals impaired kidney excretory capacity or tubular dysfunction, while increased uric acid may indicate disrupted purine metabolism and reduced renal clearance under toxic insult [59].

In our results, AP exposure produced marked elevations in serum uric acid, creatinine, and urea, suggesting compromised glomerular filtration efficiency, reduced renal clearance capacity, and potential renal tissue injury. Co-treatment with TEO alone or in combination with vitamin C attenuated these alterations, restoring biomarker levels toward control values (*p* < 0.05 vs. AP) and suggesting a protective effect against AP-induced nephrotoxicity. These findings are consistent with previous reports by Oularbi et al. [30,60], who documented renal dysfunction following lambda-cyhalothrin exposure. Similarly, thyme essential oil has been reported to exert nephroprotective effects against insecticide formulations containing chlorantraniliprole, including combined commercial formulations such as Voliam Targo^®^, mainly through its antioxidant and anti-inflammatory properties [39,47]. Vitamin C has also been shown to preserve renal function in pyrethroid-exposed rats by reducing oxidative stress and improving redox homeostasis [61].

Nephrotoxicity represents a critical consequence of pesticide exposure, given the kidney’s central role in the metabolism and excretion of xenobiotics, which renders it particularly vulnerable to toxic injury. This susceptibility is especially pronounced under conditions of repeated or prolonged exposure, such as in agricultural settings, where it may contribute to the development of chronic kidney disease (CKD) [62,63]. The structural and functional integrity of the kidney is essential for maintaining fluid and electrolyte balance, as well as metabolic homeostasis [64].

Histopathological evaluation remains a key approach for detecting organ-specific toxicity following exposure to environmental toxicants, including pesticides. In the obtained results, histological examination of kidneys from AP-treated rabbits revealed marked alterations affecting both cortical and medullary regions. Cortical lesions included glomerular atrophy, tubular degeneration, and early sclerotic changes, while the outer medulla showed vascular congestion, tubular disorganization, and increased numbers of pyknotic nuclei within collecting ducts and loops of Henle. The greater vulnerability of the cortex may be related to its higher metabolic activity and the presence of proximal tubules, whereas medullary alterations may impair the countercurrent exchange system involved in urine concentration [11].

The nephrotoxic effects of pyrethroids have been widely documented in experimental models. For instance, lambda-cyhalothrin exposure has been associated with vascular congestion, tubular degeneration, edema, and inflammatory infiltration in renal tissue [29,30,65]. These alterations are primarily attributed to oxidative stress, characterized by increased production of reactive oxygen species (ROS) and nitric oxide (NO), mitochondrial dysfunction, and depletion of antioxidant defenses [66]. Such mechanisms may lead to lipid peroxidation, DNA damage, and activation of cell death-related pathways, contributing to renal structural injury [65,66,67]. In addition to pyrethroids, emerging evidence suggests that anthranilic diamide insecticides may also contribute to renal toxicity. Although data on chlorantraniliprole are still limited, studies on related compounds such as cyantraniliprole have reported renal histopathological alterations, including tubular vacuolation and vascular congestion [68]. These findings suggest that disruption of calcium homeostasis and cellular energy balance may be involved in the observed toxicity.

Importantly, co-treatment with *Thymus vulgaris* essential oil (TEO) and/or vitamin C markedly attenuated the histopathological alterations observed in this study. This attenuation may be attributed, at least in part, to the reduction in oxidative stress and subsequent modulation of redox-sensitive signaling pathways involved in renal injury. In particular, carvacrol, the major constituent of TEO, has been reported to reduce oxidative damage and inflammatory responses in experimental models of pesticide toxicity [69,70]. In addition to its direct antioxidant activity, carvacrol may also exert nephroprotective effects through modulation of endogenous defense pathways, particularly via activation of the Keap1/Nrf2 signaling axis, which regulates cellular redox homeostasis [71,72]. Similarly, vitamin C has been shown to preserve renal structure and function in pyrethroid-exposed animals by scavenging reactive oxygen species and supporting endogenous antioxidant systems [51]. Consistent with these biochemical improvements, co-treatment was also associated with reduced histopathological damage in renal tissue. These functional impairments and histological alterations are consistent with the increased collagen deposition observed in Masson’s trichrome-stained sections of the cortical and medullary tubulointerstitium, suggesting early extracellular matrix (ECM) accumulation associated with fibrotic remodeling. This process may be linked to the oxidative stress mechanisms described above [73,74].

Oxidative stress and inflammation are closely interconnected and may contribute to the progression of renal fibrosis by inducing tubular epithelial injury and activation of interstitial fibroblasts [75]. These events are associated with activation of multiple profibrotic signaling pathways, including TGF-β/Smad, NF-κB, NOX4, PI3K, and, in some contexts, Nrf2-related responses [76,77]. Among these, TGF-β is considered a central regulator of fibrogenesis, promoting ECM accumulation, tissue remodeling, and progressive renal dysfunction through both Smad-dependent and independent mechanisms [78].

Natural compounds with antioxidant properties, including vitamins and thyme essential oils, have been reported to exert antifibrotic effects through modulation of these pathways [79,80]. In particular, vitamin C deficiency has been associated with increased expression of profibrotic markers such as α-smooth muscle actin, fibronectin, and type IV collagen via activation of TGF-β signaling [81]. Conversely, vitamin C supplementation has been shown to attenuate TGF-β/Smad signaling and reduce ECM deposition in various models of organ fibrosis [82].

Similarly, carvacrol-rich Thymus essential oils have demonstrated antifibrotic and anti-inflammatory effects in experimental models, partly through modulation of oxidative stress and TGF-β1/Smad signaling [83,84]. Previous studies have also shown that co-administration of TEO and vitamin C can mitigate Ampligo-induced oxidative stress and reduce ECM accumulation [19]. In agreement with these findings, co-treatment of rabbit kidneys was associated with a reduction in collagen deposition and attenuation of fibrotic remodeling, thereby preserving corticomedullary architecture. Mechanistically, p53 may represent a key link between pesticide-induced cellular stress and fibrotic progression [85]. In injured kidneys, p53 is often upregulated following toxic insults, promoting apoptosis, cell cycle dysregulation, and the release of profibrotic mediators such as CTGF, PAI-1, and TGF-β1. These processes may contribute to persistent inflammation, myofibroblast activation, and progression toward tubulointerstitial fibrosis and chronic kidney disease [86,87].

Consistent with this framework, our immunohistochemical findings in rabbit kidneys showed that subacute AP exposure significantly increased p53 expression compared with controls. The marked increase in tubular p53 immunoreactivity suggests activation of DNA damage and mitochondrial stress-related apoptotic pathways [85,86,88,89]. In parallel, the downregulation of the anti-apoptotic protein Bcl-2 may further contribute to enhanced cellular susceptibility to injury, reflecting an imbalance between pro- and anti-apoptotic signaling. Notably, co-treatment with TEO and/or vitamin C partially restored the p53/Bcl-2 balance altered by insecticide exposure, with the co-treatment showing the most pronounced protective effect. This response is likely associated, at least in part, with attenuation of upstream oxidative and mitochondrial stress, which are known to trigger p53 activation [29,80,89]. Consequently, the reduction in apoptosis-related alterations may contribute to limiting downstream tissue remodeling processes, including interstitial fibrosis, as suggested by the findings observed in Masson’s trichrome-stained cortical and medullary regions [41,79,90].

In the present study, AP exposure was associated with a reduction in Cyclin D1 expression, suggesting a disturbance of cell cycle regulation at the G1/S transition in renal tubular cells. Such alterations are commonly reported in toxic nephropathy and may contribute to impaired tubular repair [91]. Previous studies have shown that sustained loss of Cyclin D1 and prolonged G1/S disruption in tubular epithelium are associated with maladaptive repair processes, including epithelial disorganization and interstitial fibrosis, thereby linking defective cell cycle re-entry to the progression from acute kidney injury to chronic kidney disease [88]. In this context, the decrease in Cyclin D1 expression observed after AP exposure is consistent with the histopathological alterations identified in both cortical and medullary regions. These findings suggest a possible association between cell cycle dysregulation, p53 activation, and the expression of profibrotic mediators such as TGF-β1 and PAI-1, which are known to contribute to tubular injury and fibrosis [86,92]. In contrast, co-treatment with TEO and vitamin C was associated with partial restoration of Cyclin D1 expression, suggesting an improvement in cell cycle regulation. This effect may contribute to enhanced tubular cell recovery and reduced progression toward structural damage.

Furthermore, accumulating evidence suggests that xenobiotics may act as early triggers of tubular epithelial injury, with disruption of cell–cell adhesion and junctional integrity representing a key initiating event. Prozialeck and Edwards [93] reported that nephrotoxic injury is frequently associated with alterations in epithelial adhesion systems and tight junction architecture. In particular, cadherins and catenins, together with tight junction proteins such as ZO-1, occludin, and claudins, play essential roles in maintaining tubular barrier function and paracellular permeability. In addition, adhesion-related pathways involving ICAM-1, integrins, and selectins may contribute to leukocyte recruitment and amplification of inflammatory responses during nephrotoxic injury [93,94,95]. Experimental studies have also highlighted the involvement of Wnt/β-catenin signaling in renal injury and fibrosis. Activation of this pathway in tubular epithelial cells has been associated with epithelial–mesenchymal crosstalk and fibrogenic responses, partly mediated by β-catenin-dependent transcriptional programs [96].

In the present study, AP exposure was associated with decreased E-cadherin expression and altered β-catenin localization in renal tissues, suggesting alterations of adherens junctions and epithelial integrity. These alterations may be related to stress-induced signaling pathways, including TGF-β-mediated regulation of epithelial markers, which has been reported to reduce E-cadherin expression and promote mesenchymal features such as vimentin and α-smooth muscle actin [97,98]. In parallel, activation of Wnt/β-catenin signaling may contribute to cytoplasmic and nuclear accumulation of β-catenin, thereby promoting the expression of fibrosis-related genes, including collagens and fibronectin [95,99].

Importantly, co-treatment with TEO and vitamin C was associated with partial normalization of E-cadherin and β-catenin expression patterns, suggesting preservation of epithelial junction integrity. This effect may be linked, at least in part, to attenuation of oxidative stress and modulation of signaling pathways such as TGF-β and Wnt/β-catenin. Similar protective effects of antioxidant interventions on epithelial markers and tissue structure have been reported in other models of pesticide-induced toxicity [41]. Likewise, Khaldoun et al. [38] reported improved E-cadherin and β-catenin immunoreactivity in intestinal tissues of rabbits exposed to the combined insecticide formulation Voliam Targo^®^ following treatment with carvacrol-rich TEO, highlighting the potential contribution of its antioxidant and anti-inflammatory properties [70].

## 5. Conclusions

Subacute exposure to Ampligo^®^ 150 ZC induced significant renal alterations in rabbits, including biochemical disturbances, histopathological damage, collagen deposition, dysregulation of apoptosis- and cell cycle-related markers, and disruption of epithelial integrity. These findings indicate that AP-induced nephrotoxicity likely involves oxidative stress, apoptosis, and epithelial dysfunction. Co-treatment with carvacrol-rich thyme essential oil and vitamin C, particularly in combination, markedly attenuated these effects and improved renal structure and function. These protective effects are likely mediated through antioxidant, anti-inflammatory, anti-apoptotic, and antifibrotic properties.

Overall, these results highlight the nephrotoxic potential of this pesticide formulation and support the use of thyme essential oil and vitamin C as complementary protective agents. Further research is warranted to evaluate long-term outcomes under chronic or occupational exposure conditions.

## 6. Limitations of This Study

While this study provides robust multilevel evidence of Ampligo^®^ 150 ZC-induced nephrotoxicity and TEO + vitamin C renoprotection in female rabbits, several limitations should be acknowledged. The 28-day subacute exposure regimen (20 mg/kg, every other day) effectively captures early pathological mechanisms but does not reflect chronic occupational exposures relevant to agricultural workers. Additionally, the female-only design precludes assessment of sex-based differences in susceptibility and response. Finally, while histopathological and immunohistochemical analyses strongly suggest underlying protective mechanisms, the absence of direct biochemical assays (ROS, MDA, GSH, SOD/CAT) limits definitive mechanistic confirmation. Additional investigations should incorporate chronic exposure models, include both sexes, and apply integrative approaches such as multi-omics analyses.

## Figures and Tables

**Table 1:** Effects of AP, AP + TEO, and AP + TEO + vitamin C on body weight and on absolute and relative kidney weights in female rabbits during the acclimatization period and after 14 and 28 days of treatment. * *p* < 0.05; ** *p* < 0.01 compared with the control group, ^#^ *p* < 0.05; ^##^ *p* < 0.01; compared with female rabbits treated with AP group.

	Period/Group		Control	AP	AP + TEO	AP + TEO + VitC
Body weight (kg)	Acclimatization	Day 1	2.27 ± 0.12	1.92 ± 0,36	2.14 ± 0.17	2.17 ± 0.19
		Day 14	2.61 ± 0.06	2.50 ± 0.08	2.43 ± 0.07	2.51 ± 0.09
	Experimentation	Day 14 Day 28	3.01 ± 0.07 3.30 ± 0.06	2.54 ± 0.10 * 2.77 ± 0.06 **	2.76 ± 0.02 2.92 ± 0.06	2.87 ± 0.10 3.23 ± 0.07 ^##^
Kidney weight (g)	Right kidney	Absolute weight	9.50 ± 1.79	8.20 ± 0.19 **	9.18 ± 0.66 ^#^	9.50 ± 0.84 ^##^
		Relative weight	3.75 ± 0.46	2.59 ± 0.23 **	3.21 ± 0.49 ^#^	3.55 ± 0.40 ^##^
	Left kidney	Absolute weight	9.33 ± 1.13	8.00 ± 0.34 **	9.17 ± 1.45 ^##^	9.39 ± 0.98 ^##^
		Relative weight	3.68 ± 0.36	2.23 ± 0.22 **	3.20 ± 0.36 ^#^	3.38 ± 0.16 ^##^

**Table 2:** Effects of thyme essential oil and vitamine C on Ampligo^®^-Induced enhancement of the renal function biomarkers in female rabbits. Data are expressed as means ± S.D. (*n* = 5 rabbits for each group).* *p* < 0.05; ** *p* < 0.01 compared with the control group, # *p* < 0.05 compared with femel rabbits treated with the AP group.

Experimental Groups	BUN	Creatinine	Uric Acid
Control	00.229 ± 0.02	08.80 ± 0.17	0.25 ± 0.09
AP	00.44 ± 0.06 **	10.27 ± 0.37 *	01.32 ± 0.40 **
AP + TEO	00.30 ± 0.02 ^#^	08.96 ± 0.41 ^#^	00.55 ± 0.34 ^#^
AP + TEO + Vit C	00.29 ± 0.04 ^#^	08.72 ± 0.52 ^#^	00.45 ± 0.16 ^#^

## Data Availability

The original contributions presented in this study are included in the article/Supplementary Material. Further inquiries can be directed to the corresponding author.

## Supplementary Materials

The following supporting information can be downloaded at: https://www.mdpi.com/article/10.3390/jox16030074/s1, Figure S1: Protective effects of Thyme Essential Oil (TEO) and/or Vitamin C on histopathological alterations induced by Ampligo exposure in renal cortex rabbit tissue sections; Figure S2: Protective effects of Thyme Essential Oil (TEO) and/or Vitamin C on histopathological alterations induced by Ampligo exposure in renal medulla rabbit tissue sections; Figure S3: Masson’s trichrome staining showing collagen deposition in the cortical and medullary regions of rabbit kidneys from the different experimental groups; Figure S4: Immunohistochemical staining of p53 in the cortical and medullary regions of female rabbit kidneys; Figure S5: Immunohistochemical staining of Bcl-2 in the cortical and medullary regions of female rabbit kidneys; Figure S6: Immunohistochemical staining of Cyclin D1 in the cortical and medullary regions of female rabbit kidneys; Figure S7: Immunohistochemical staining of E-cadherin in the cortical and medullary regions of female rabbit kidneys; Figure S8: Immunohistochemical staining of β-catenin in the cortical and medullary regions of female rabbit kidneys.

## Abbreviations

The following abbreviations are used in this manuscript:

AP	Ampligo
TEO	Thyme Essential Oil
Vit C	Vitamin C
