# Supplementary material for: Apoptosis and Cell Cycle Dysregulation in Ampligo® 150 ZC-Induced Nephrotoxicity in Female Rabbits: Protective Effects of Thymus vulgaris Essential Oil and Vitamin C
Source: J Xenobiot. 2026 Apr 27;16(3):74. doi: 10.3390/jox16030074 (PMC13214766; doi:10.3390/jox16030074)

Journal of Xenobiotics

MDPI

# Apoptosis and cell cycle dysregulation in Ampligo® 150 ZC-induced nephrotoxicity in female rabbits: Protective effects of Thymus vulgaris essential oil and vitamin C

**Louisa Bechohra, Chahrazed Makhlouf, Hassina Khaldoun, Samira Aouichat, Amina Settar, Dalila Tarzaali, Nacera Lemlikchi, Amina Bouhallel, Yasmine Oularbi, Schahinez Terkmane, Nacima Djennane**

Figure S1: Protective effects of Thyme Essential Oil (TEO) and/or Vitamin C on histopathological alterations induced by Ampligo exposure in renal cortex rabbit tissue sections.

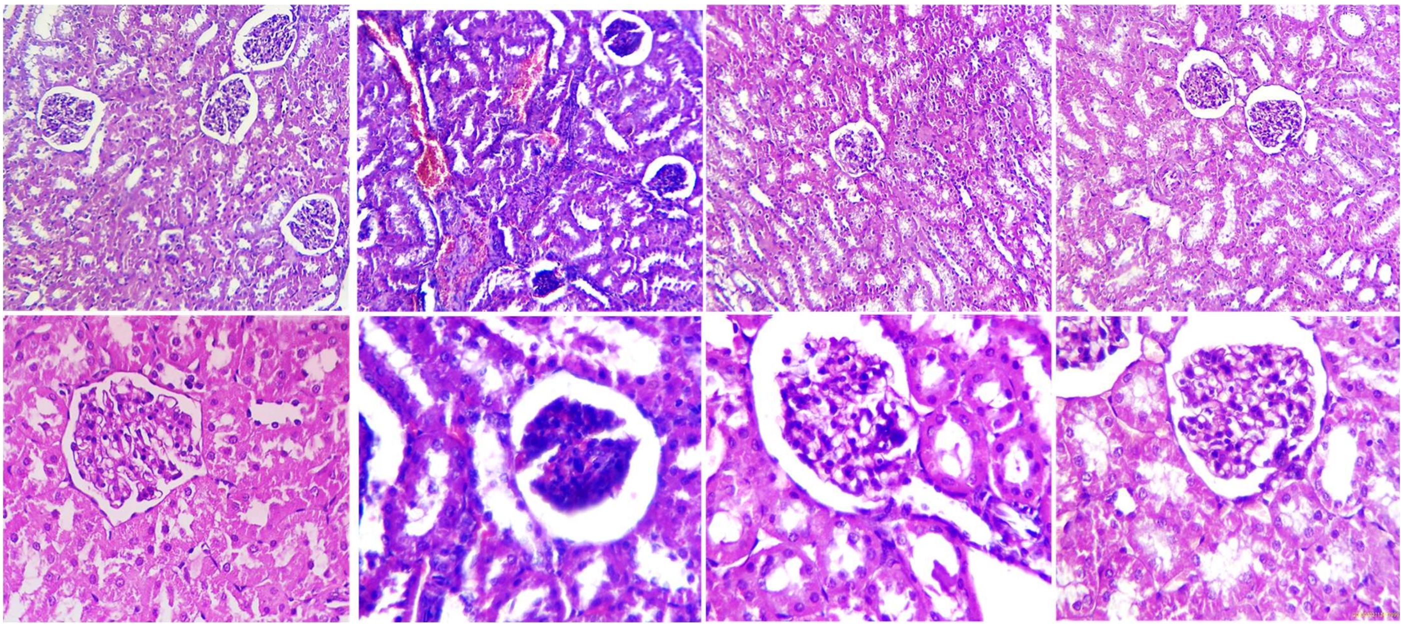

Figure S2: Protective effects of Thyme Essential Oil (TEO) and/or Vitamin C on histopathological alterations induced by Ampligo exposure in renal medulla rabbit tissue sections.

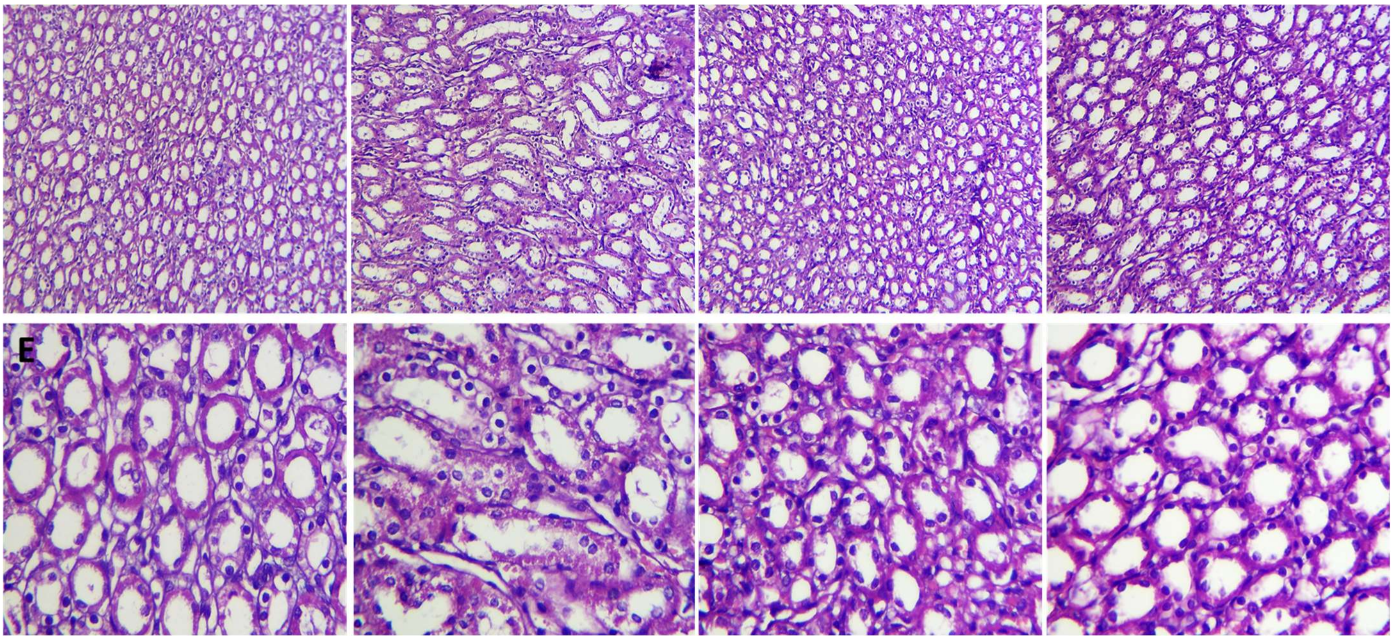


Figure S3: Masson's trichrome staining showing collagen deposition in the cortical and medullary regions of rabbit kidneys from the different experimental groups

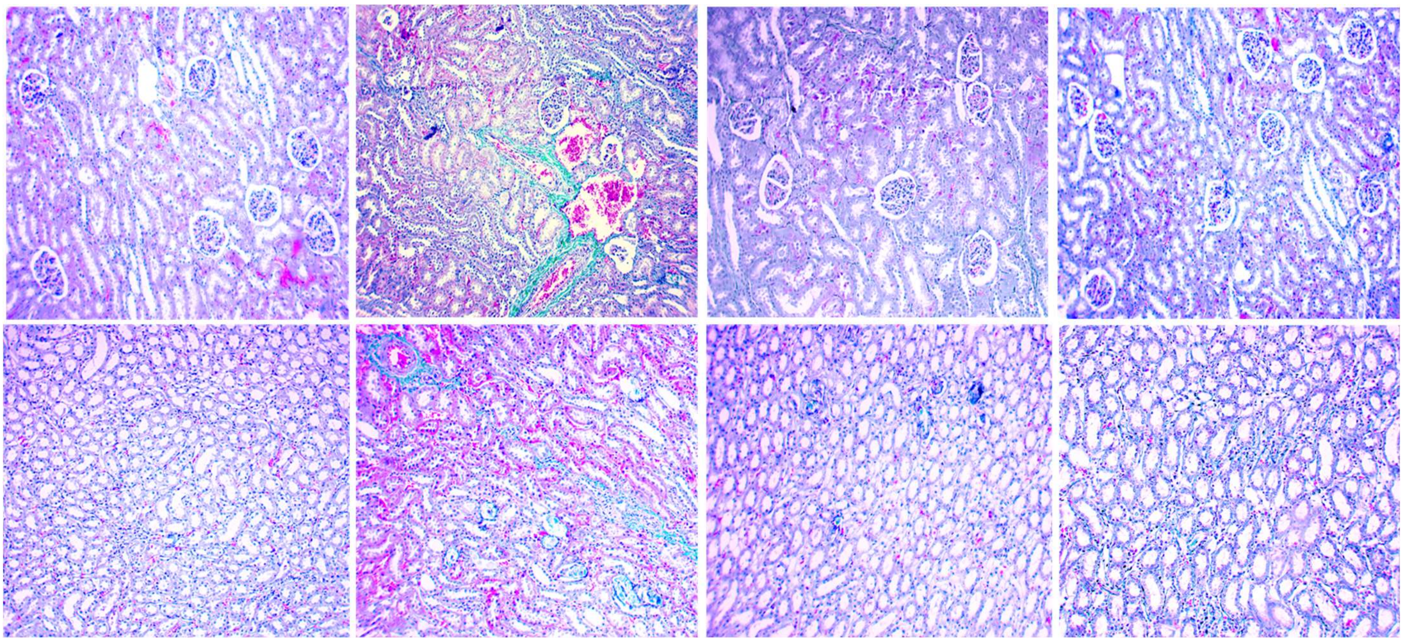

Figure S4: Immunohistochemical staining of p53 in the cortical and medullary regions of female rabbit kidneys.

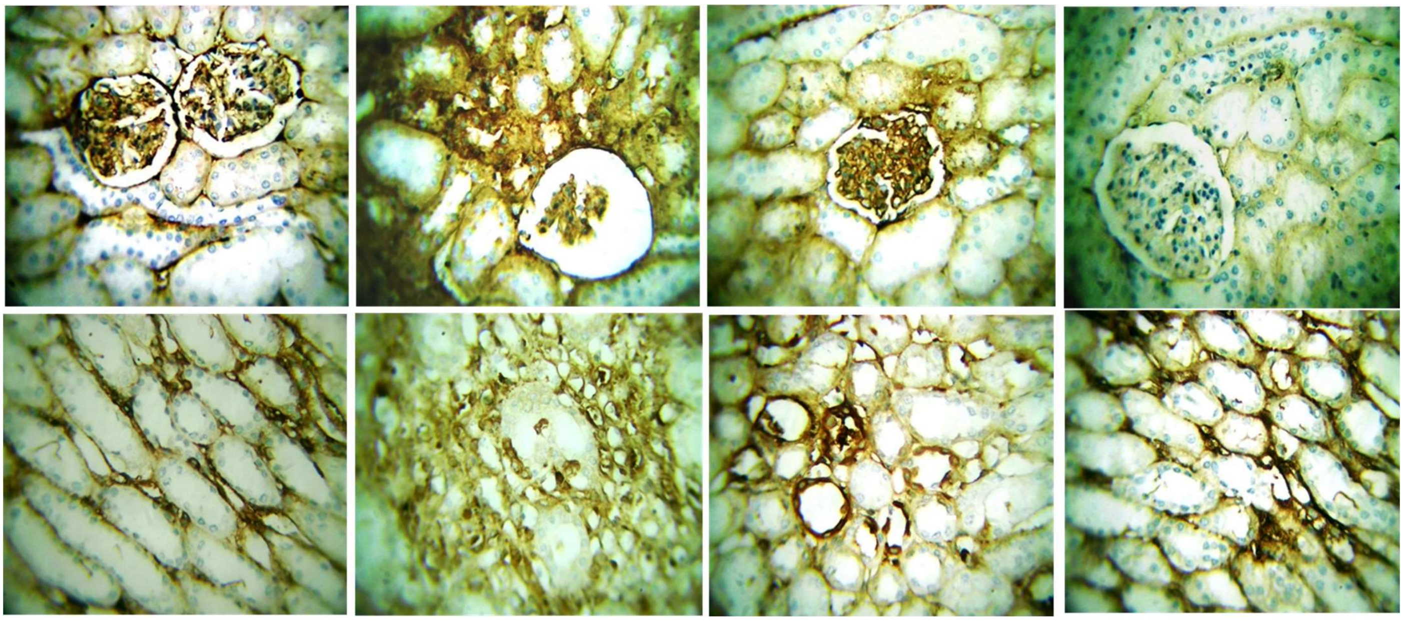

Figure S5: Immunohistochemical staining of Bcl-2 in the cortical and medullary regions of female rabbit kidneys.

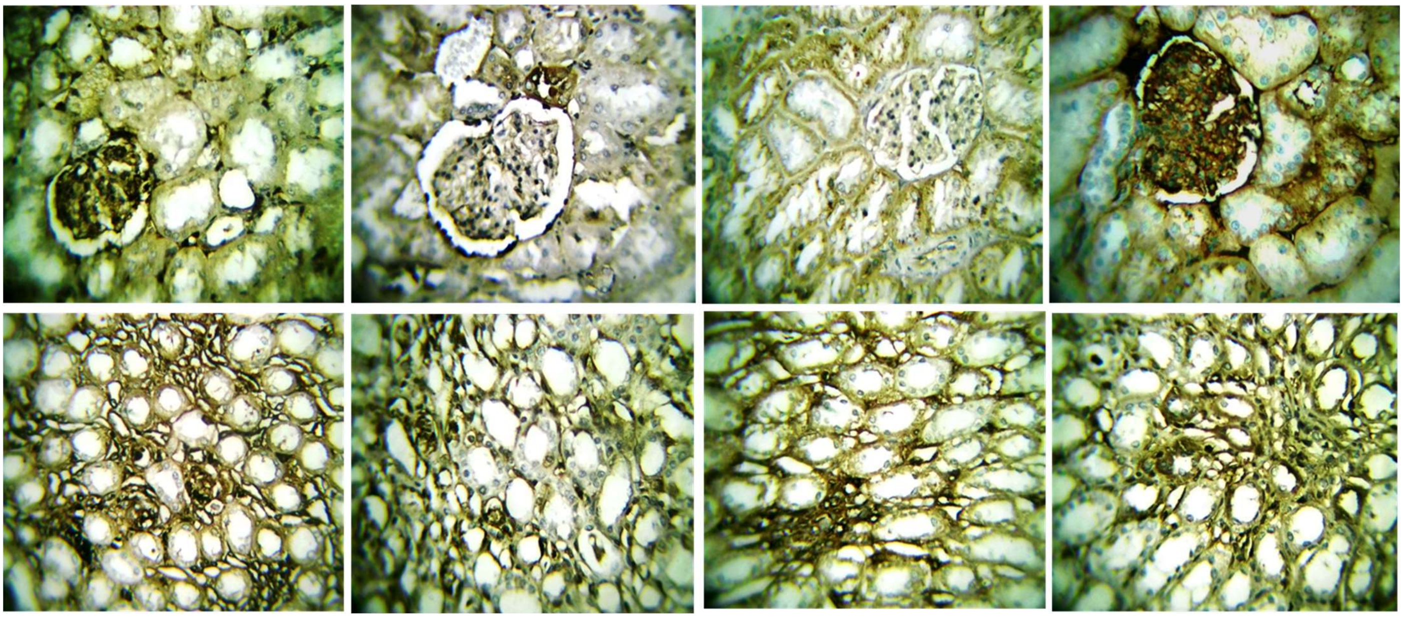

Figure S6: Immunohistochemical staining of Cyclin D1 in the cortical and medullary regions of female rabbit kidneys.

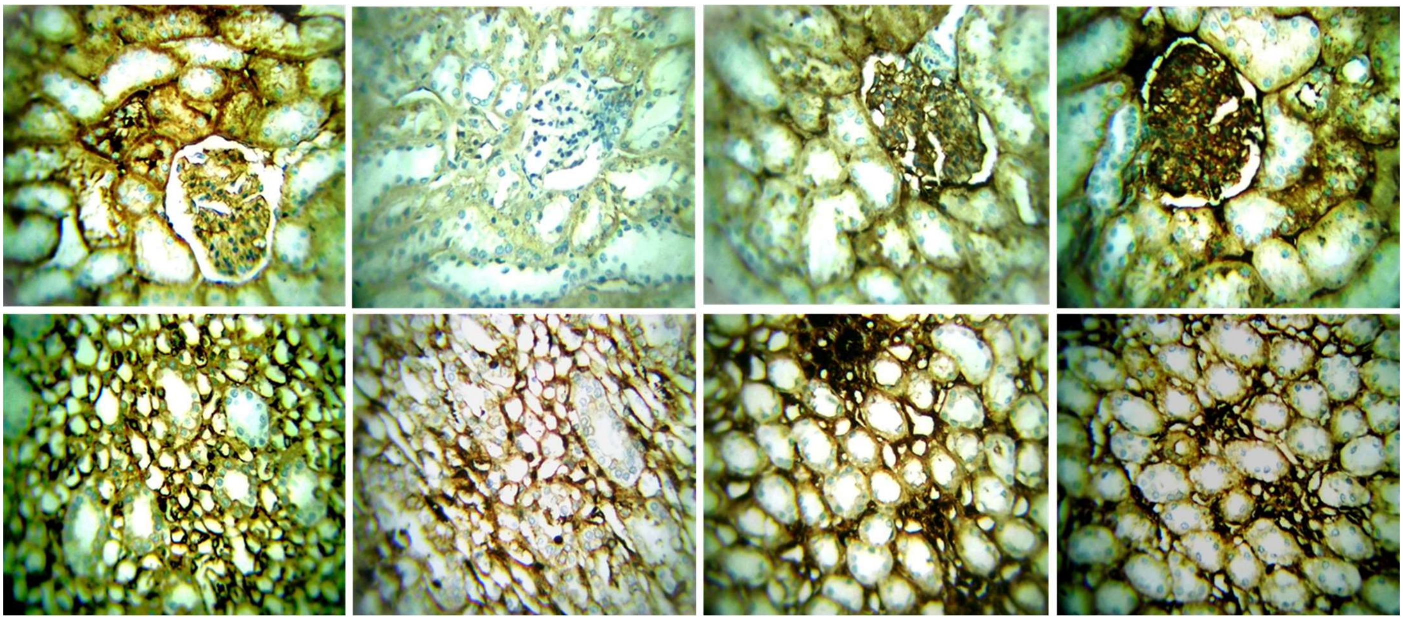

Figure S7: Immunohistochemical staining of E-cadherin in the cortical and medullary regions of female rabbit kidneys.

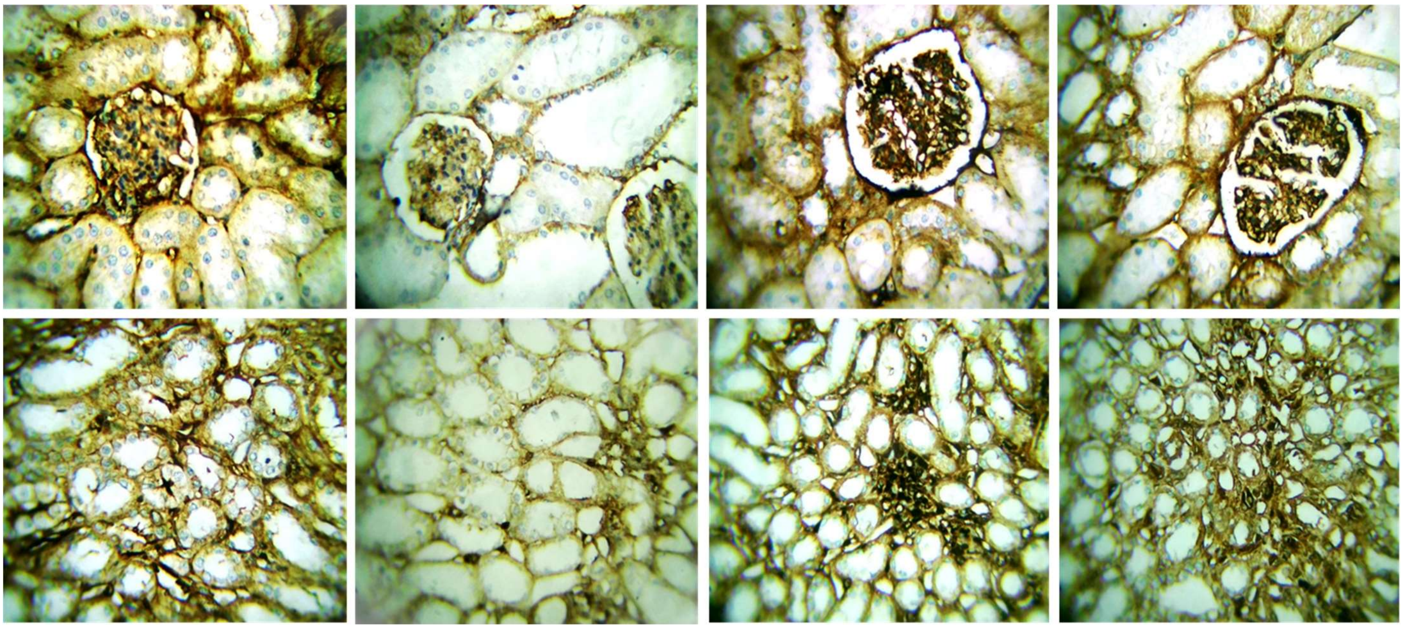

Figure S8: Immunohistochemical staining of β-catenin in the cortical and medullary regions of female rabbit kidneys

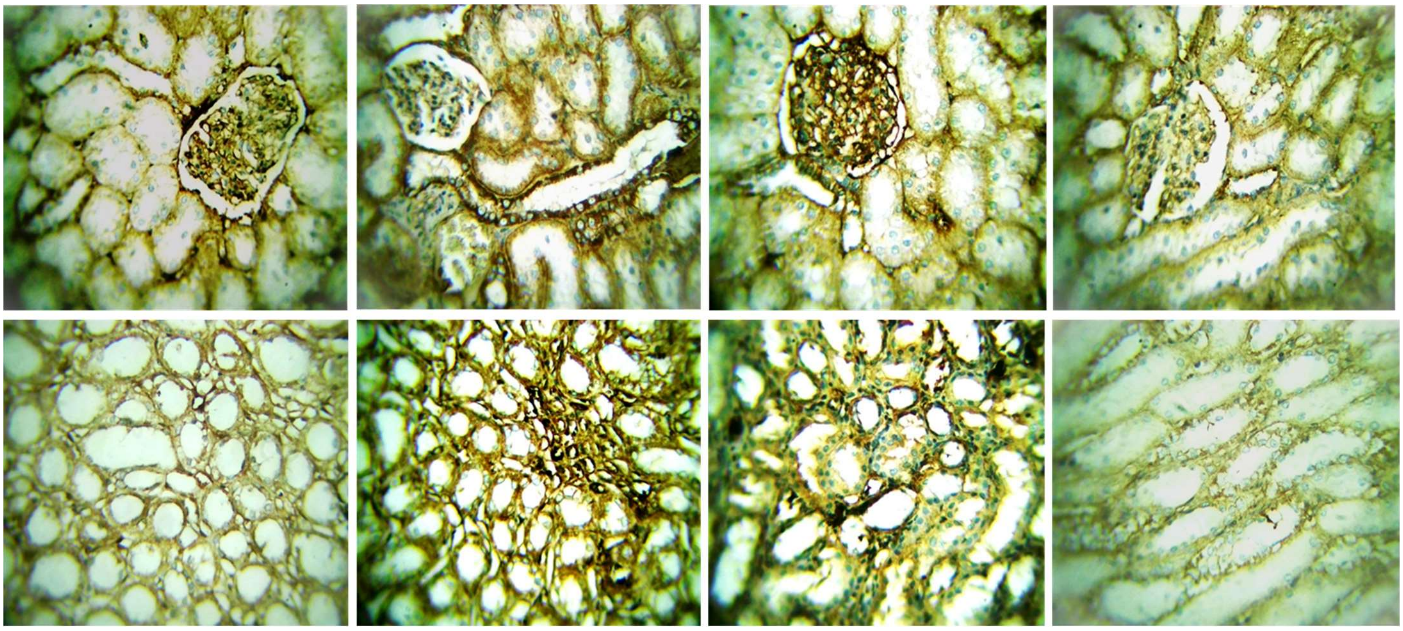

Supplement: Supplementary file 1 [file jox-16-00074-s001.zip › jox-4228736-supplementary.pdf]
